# Appendiceal mixed adenoneuroendocrine carcinomas, a rare entity that can present as a Krukenberg tumor: case report and review of the literature

**DOI:** 10.1186/s12957-015-0740-1

**Published:** 2015-11-26

**Authors:** Margarita Romeo, Ariadna Quer, Antoni Tarrats, Carlos Molina, Joaquim Radua, José-Luís Manzano

**Affiliations:** Medical Oncology Department, Institut Català d’Oncologia Badalona (Hospital Germans Trias i Pujol), Carretera del Canyet s/n, 08916 Badalona, Barcelona Spain; Pathology Department, Hospital Germans Trias i Pujol, Carretera del Canyet s/n, 08916 Badalona, Barcelona Spain; Gynecology Department, Gynecologic Oncology Section, Hospital Germans Trias i Pujol, Carretera del Canyet s/n, 08916 Badalona, Barcelona Spain; Gynecology Department, Hospital de l’Esperit Sant, Av. Mossèn Pons i Rabadà, s/n, 08923 Santa Coloma de Gramanet, Barcelona Spain; King’s College London, 16 De Crespigny Park, SE5 8AF London, UK; FIDMAG Germanes Hospitalàries—CIBERSAM, Dr. Antoni Pujadas, 38, 08830 Sant Boi de Llobregat, Barcelona Spain

**Keywords:** Goblet cell carcinoid, Adenoneuroendocrine carcinoma, Appendix, Adenocarcinoid, Krukenberg tumor

## Abstract

**Background:**

Mixed adenoneuroendocrine carcinoma is a rare tumor recently recognized as a new category in the last World Health Organization (WHO) classification of appendiceal tumors (2010). This term has been proposed to designate carcinomas of the appendix that arise by progression from a pre-existing goblet cell carcinoid. Mixed adenoneuroendocrine carcinomas are more aggressive tumors than typical goblet cell carcinoids and usually present with peritoneal spreading and ovarian masses. Staging, some histological features, and completeness of surgery are factors that determine its evolution.

**Case Presentation:**

We report the case of a mixed adenoneuroendocrine carcinoma—signet ring cell subtype—that presented as a Krukenberg tumor of unknown primary.

**Conclusion:**

The review of literature is focused on the most recent WHO pathologic classification of appendiceal tumors containing goblet cell clusters, which seems to correlate with prognosis. A management proposal for mixed adenoneuroendocrine carcinomas reported in previous literature is also discussed. This ranges from right hemicolectomy to cytoreduction plus hyperthermic intraperitoneal chemotherapy, in both cases usually followed by intravenous chemotherapy.

## Background

Mixed appendiceal adenoneuroendocrine carcinoma is an extremely rare tumor recently recognized as an independent entity in the last World Health Organization (WHO) classification of appendiceal tumors, which dates from 2010. This term has been proposed to designate carcinomas of the appendix that arise by progression from a pre-existing goblet cell carcinoid. Morphologically, it is composed of goblet cell clusters and carcinoma cells, each component representing at least 30 % of the tumor, showing important cytologic atypia [1].

Appendiceal malignant epithelial tumors account for 1 % approximately of all gastrointestinal neoplasms and are mainly divided in neuroendocrine neoplasms and carcinomas [2]. In the past, goblet cell carcinoids, or formerly called “adenocarcinoids,” were considered a category of the neuroendocrine neoplasms that included all neoplasms containing goblet cell clusters [3]. However, the disparity of pathologic features and prognosis leads Tang et al. in 2008 to propose a subclassification of this category and to propose a differential therapeutic algorithm [4]. Based on this research, the 2010 WHO classification of appendiceal tumors recognized two separate entities, both considered to be neuroendocrine tumors: classic goblet cell carcinoids (GCCs) and mixed adenoneuroendocrine carcinomas.

Goblet cell clusters, which are the neuroendocrine distinctive component of these two tumors, are small nests of signet ring-like cells resembling normal intestine goblet cells except for nuclear compression and atypia. These clusters show a characteristic submucosal growth that affects the base of crypts and presents positivity for both neuroendocrine and epithelial markers. While GCCs (definition from 2010 WHO classification) are composed of well-defined goblet cell clusters with mild to moderate atypia, mixed adenoneuroendocrine carcinomas are considered more aggressive tumors. These are characterized by significant atypia, partial loss of goblet cell clusters, and presence of carcinoma cells with a wide range of differentiation (from signet ring cell—SRC—to poorly differentiated adenocarcinoma), without apparently any neoplastic change in the mucosa [1, 4].

This report presents the case of a Krukenberg tumor originated from an adenoneuroendocrine carcinoma and reviews the literature about this extremely infrequent histology and its management.

## Case presentation

**Fig. 1:**
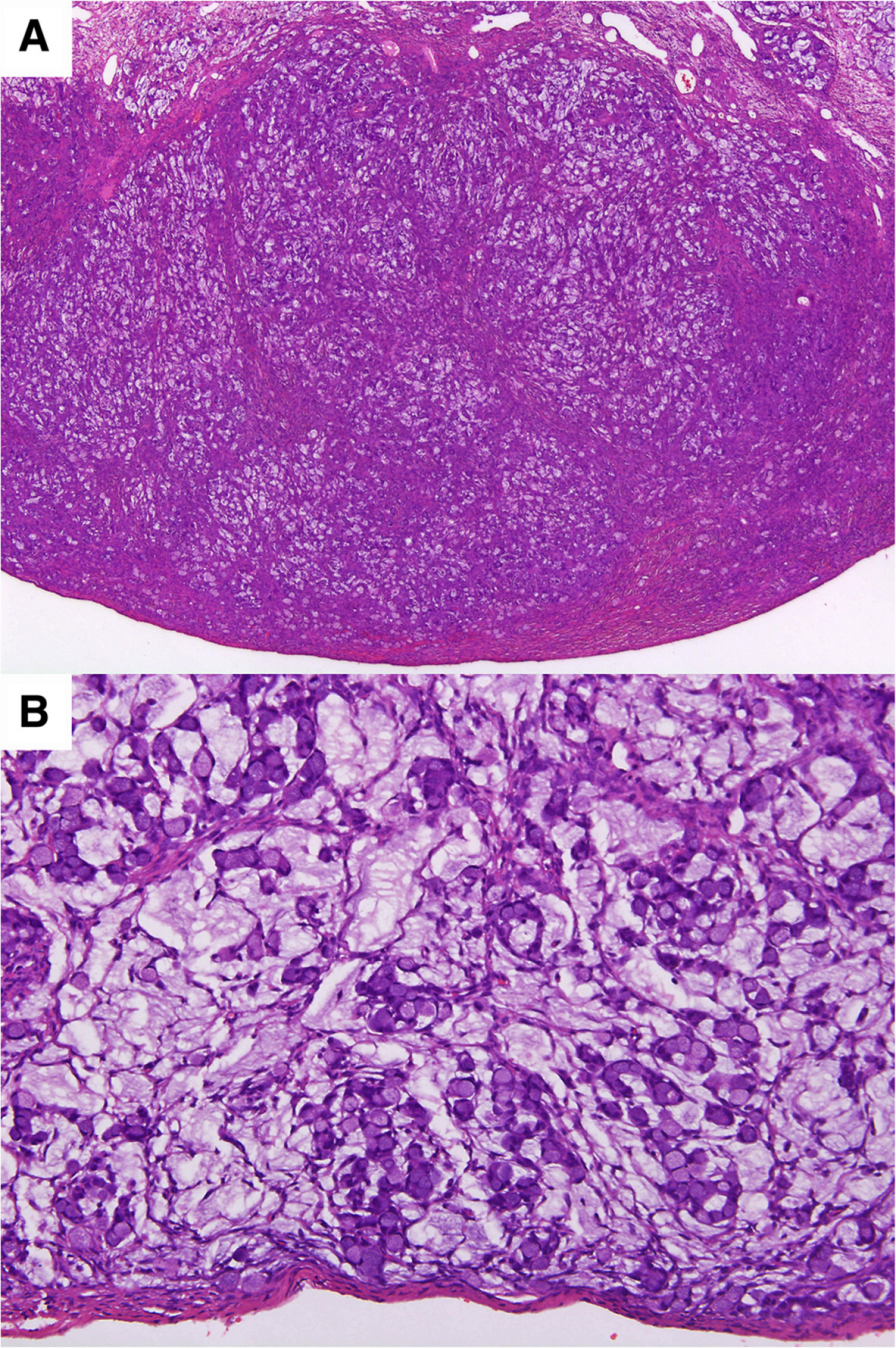
Ovarian masses. **a** Ovarian stroma. **b** Aggregates of mucin

**Fig. 2:**
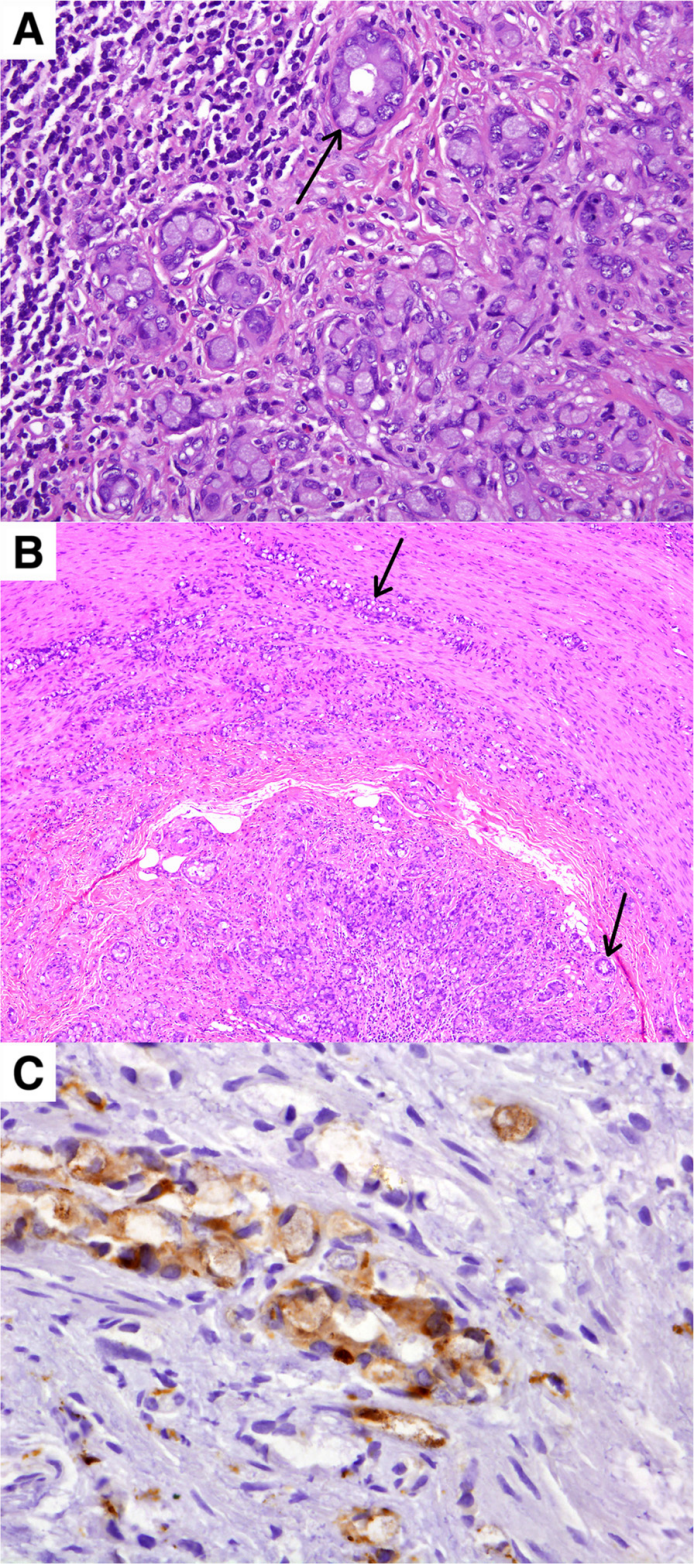
Appendiceal tumor. **a** Significant cytologic atypia. **b** Destruction of the appendiceal wall. **c** Focal immunoreactivity for synaptophysin

A 75-year-old woman without significant medical history and an excellent performance status was diagnosed of a 87 × 73-mm solid right ovarian mass during a gynecologic review. CA125, CA 199, and CEA were normal. A CT scan excluded distant lesions. Laparoscopy revealed a white, encapsulated ovarian mass, resembling a thecoma, without extraovarian implants. Hysterectomy and double adnexectomy were performed. Pathologists reported a SRC adenocarcinoma, invading all genital organs and showing prominent lymphovascular permeation. Tumor growth in the ovarian stroma exhibited a nodular pattern (see Fig. 1a) and irregular aggregates of mucin (Fig. 1b), suggesting a mucinous Krukenberg tumor. Tumor cells were diffusely positive for CK 20, focally positive for CK7, and negative for estrogen receptors. Peritoneal washings were negative for malignant cells. Several complementary exams were performed but failed to detect an extraovarian primary (fibrocoloscopy, fibrogastroscopy, PET-CT, mammographies). Two months later, the patient remained asymptomatic, and appendectomy was planned to exclude a primary appendiceal tumor. During this procedure, an appendiceal mass and multiple peritoneal implants were discovered. A complete cytoreductive surgery and right hemicolectomy (RH) were finally performed. Pathologists reported an appendiceal adenoneuroendocrine carcinoma—SRC type—invading peritoneum and one paraaortic lymph node. Key points in the resected appendix, for pathologic diagnosis, were the presence of goblet cell clusters and SRC arranged in irregular large clusters, single-cell infiltrating pattern and significant cytologic atypia (Fig. 2a); destruction of the appendiceal wall (Fig. 2b); and focal immunoreactivity for synaptophysin (Fig. 2c). Adjuvant chemotherapy was delivered (FOLFOX, 12 cycles). After 2 years, the patient is currently free of relapse.

### Discussion

Mixed appendiceal adenoneuroendocrine carcinomas and GCCs have been historically included in the same category (referred as “adenocarcinoids” or simply “GCCs”) because of the presence of goblet cell clusters, despite the prognosis disparity reported in historical series. Early studies of adenocarcinoids, the majority of them reported in the 1970s and 1980s, pointed to their intermediate features and prognosis between typical appendiceal carcinoids and colonic adenocarcinomas, as well as their spectrum of goblet cell cluster percentage, mucine amount, nuclear atypia, mitotic count, and metastatic ability [5–12]. A review of 57 studies (including nearly 600 patients) found that 5-year overall survival ranged between 60 and 84 % [13].

Present 2010 WHO pathologic classification of appendiceal tumors differentiates GCC from adenoneuroendocrine carcinomas [1] based on morphologic criteria developed by Tang et al., which were reported in 2008 with high interobserver concordance [4]. The authors classified 63 cases of appendiceal adenocarcinoids collected from Memorial Sloan Kettering Cancer Center between 1993 and 2005 into three categories: typical GCC (group A, 30 cases), adenocarcinoma ex GCC subtype SRC (group B, 26 cases), and adenocarcinoma ex GCC subtype poorly differentiated (group C, 7 cases). Group A tumors presented well-defined goblet cell clusters with mild to moderate atypia. Group B tumors were composed of goblet cells and SRC arranged in irregular large clusters with partial loss of typical goblet cell cluster architecture, significant atypia, single-cell infiltrating pattern, and destruction of the appendiceal wall. Group C tumors’ hallmark was the presence of at least one foci that could not be distinguished from a poorly differentiated adenocarcinoma or undifferentiated carcinoma. Extracellular mucin could be prominent in groups A and B tumors. Remarkably, PET performed to our patient had not detected peritoneal disease, probably due to the significant mucinous component of the tumor. Additionally, all groups presented focal immunoreactivity for neuroendocrine markers (chromogranin or synaptophysin mainly); however, while groups A and B showed normal expression of intestinal mucin glycoproteins (MUC1−/MUC2+) and Ki67 < 20 %, group C had abnormal p53 expression, MUC1+/MUC2− pattern, and Ki67 > 70 %. Previously, Alsaad had described strong immunoreactivity for CK20 and inconsistent immunoreactivity for CK7 in a series on 17 appendiceal goblet cell-containing tumors, similarly to colonic adenocarcinomas [14]. In the 2010 WHO classification, group A is referred as GCC and groups B and C are referred to as mixed adenoneuroendocrine carcinomas.

Both GCC and mixed adenoneuroendocrine carcinomas are thought to arise from a crypt base stem cell that will develop both neuroendocrine and glandular differentiation [15]. Adenoneuroendocrine carcinomas are considered to be more aggressive forms developed from pre-existing GCC. Interestingly, discordant histology between primary appendiceal adenocarcinoids and their peritoneal implants was reported in nine cases by Yan et al. in 2008 [16]. Peritoneal implants from these patients had lost the neuroendocrine component and goblet cell clusters, as ovarian metastases in our case (Fig. 1). This phenomenon is consistent with progression of GCC to the more aggressive mixed adenoneuroendocrine carcinomas.

Median age of presentation of GCC and appendiceal adenoneuroendocrine carcinomas ranges between the fifth and sixth decades of life. The most common clinical presentation is acute appendicitis (nearly or above 50 % of cases). Other symptoms are abdominal pain, abdominal mass, bowel obstruction, gastrointestinal bleeding, or incidental finding [4, 6, 8, 10, 13, 17–20]. Tang et al. found that abdominal pain plus lower abdominal palpable mass was the most frequent presentation (50 %), followed by acute appendicitis (44 %) [4]. In a series of 16 appendiceal adenocarcinoids diagnosed between 1995 and 2005 in Mount Sinai Hospital, ten tumors had been found incidentally [21].

Staging is based on the colonic adenocarcinomas staging system from American Joint Committee on Cancer (AJCC) [18, 22]. In the series reported by Tang et al., pathologic classification correlated with staging and prognosis. While 70 % of group A tumors presented with stage I or II, 90 % of groups B and C presented with stage IV and mesenteric nodal disease. Stages I and II achieved an excellent survival after surgery with or without chemotherapy. However, 5-year overall survival of patients with stage IV disease was 100, 38, and 0 % for groups A, B, and C, respectively [4].

Ovaries and peritoneum are the most common metastatic sites for mixed adenoneuroendocrine carcinomas [4, 6, 8, 10, 13, 17, 18, 21]. Up to 83 % of women with stage IV disease present with ovarian masses [4]. Therefore, mixed appendiceal adenoneuroendocrine carcinomas should be considered as possible origins of Krukenberg tumors, whose definition is “ovarian metastases from mucinous tumors with at least 10 % of SRC component” [23, 24]. Distinction from a primary ovarian tumor can be difficult because some primary ovarian tumors can also contain SRC [25]. Significant expansion of the ovarian stroma due to edema, fibrosis, or cell proliferation is the main feature of Krukenberg tumors, providing a macroscopic appearance similar to our patient’s ovarian mass [26]. Krukenberg tumors account for 30–40 % of ovarian metastases [27]. Usual primaries are gastrointestinal, biliary-pancreatic, or breast [23]. Characteristics that suggest an extraovarian origin rather than a primary ovarian neoplasm are bilateralism, small size, nodular appearance, heterogeneity, destructive stromal invasion, surface implants, and lymphovascular permeation [25]. Immunochemistry may provide additional information. The pattern “diffuse CK20+/focal CK7+” of our case suggested an appendiceal origin [26]. Up to two thirds of Krukenberg tumors are diagnosed without clinical evidence of the primary before a surgical procedure [24]. Indeed, appendectomy is recommended in cases of Krukenberg tumors of unknown primary. In a series reported in 2007 of 30 cases of resected appendiceal tumors and ovarian metastases with “GCC-like and SRC pattern,” diagnosed synchronous or metachronous, most appendixes did not present a macroscopically measurable tumor [28].

Management of GCC and mixed adenoneuroendocrine tumors has been historically extrapolated from treatment of appendiceal adenocarcinomas, for which RH (with excision of regional lymph nodes) has long been considered a standard [29, 30]; however, this issue has importantly evolved in the last decade. Tang et al. recommend customizing treatments to the new classification [4]. Firstly, for pT1/pT2 typical GCC with negative margins, only surgery (appendectomy or RH) is proposed as the standard of care. Reported retrospective studies of “adenocarcinoid tumors” suggest that RH does not provide a survival benefit over appendectomy (plus en bloc removal of mesoappendix) in some selected patients [18, 21]; a meta-analysis of retrospective data from 100 patients with “appendiceal adenocarcinoid” supports the use of appendectomy as the preferred option for localized low-grade tumors with negative margins and non-invaded base of appendix [31]. Secondly, in case of SRC adenoneuroendocrine carcinomas, pT3/pT4, perforated tumors, or positive margins in the appendectomy, RH is the recommended option, followed by intravenous—IV—chemotherapy in stage III disease. And thirdly, in cases of poorly differentiated adenoneuroendocrine carcinomas or in cases with intraperitoneal spread, cytoreduction plus hyperthermic intraperitoneal chemotherapy—HIPEC—followed by IV chemotherapy [4] is the recommended option by these researchers. HIPEC is arising as a new option for neoplasms with frequent peritoneal dissemination, which is the main cause of death of these patients. Whether performing (or not) HIPEC in our 75-year-old patient is a controversial issue due to morbidity. Finally, taking into account the high incidence of ovarian metastases from GCC and mixed adenoneuroendocrine carcinomas, some authors also recommend prophylactic oophorectomy when resecting the primary tumor [4, 10, 13, 18].

Little evidence supports the use of IV chemotherapy, though several case reports using different drugs are published. Usual schemes are those used for colonic adenocarcinomas, which include 5FU, leucovorin, oxaliplatin, or irinotecan [4, 13, 18, 29]. Similarly to typical neuroendocrine carcinomas, schemes with cisplatin plus etoposide or 5-fluorouracil, cisplatin, and streptozotocin have also been used [17].

## Conclusions

In conclusion, and according to the last WHO classification of gastrointestinal neoplasms, appendiceal tumors containing goblet cell clusters are divided into GCC and mixed adenoneuroendocrine carcinomas. The latter can contain SRC or poorly differentiated carcinoma cells. This classification correlates with staging and prognosis. Mixed adenoneuroendocrine carcinomas behave more aggressively than classic GCC and usually present with peritoneal spreading and ovarian masses. A possible presentation may be as a Krukenberg tumor of unknown origin; in this case, appendectomy may be crucial to discover the primary. Their management may range from RH to cytoreduction with HIPEC, usually followed by IV chemotherapy.

## Consent

Written informed consent was obtained from the patient for publication of this case report and any accompanying images. A copy of the written consent is available for review by the Editor-in-Chief of this journal.

## Abbreviations

GCC: goblet cell carcinoid
HIPEC: hyperthermic intraperitoneal chemotherapy
IV: intravenous
RH: right hemicolectomy
SRC: signet ring cell
WHO: World Health Organization
